# Containing the accidental laboratory escape of potential pandemic influenza viruses

**DOI:** 10.1186/1741-7015-11-252

**Published:** 2013-11-28

**Authors:** Stefano Merler, Marco Ajelli, Laura Fumanelli, Alessandro Vespignani

**Affiliations:** 1 Bruno Kessler Foundation, Trento, Italy; 2 Department of Mathematics, University of Trento, Trento, Italy; 3 Laboratory for the Modeling of Biological and Socio-technical Systems, Northeastern University, Boston 02115, MA, USA; 4 Computational Epidemiology Laboratory, Institute for Scientific Interchange (ISI), Torino, Italy; 5 Institute for Quantitative Social Sciences at Harvard University, Cambridge, MA 02138, USA

**Keywords:** BSL Laboratory, Influenza, Agent-based model, Outbreak containment, Contact tracing

## Abstract

**Background:**

The recent work on the modified H5N1 has stirred an intense debate on the risk associated with the accidental release from biosafety laboratory of potential pandemic pathogens. Here, we assess the risk that the accidental escape of a novel transmissible influenza strain would not be contained in the local community.

**Methods:**

We develop here a detailed agent-based model that specifically considers laboratory workers and their contacts in microsimulations of the epidemic onset. We consider the following non-pharmaceutical interventions: isolation of the laboratory, laboratory workers’ household quarantine, contact tracing of cases and subsequent household quarantine of identified secondary cases, and school and workplace closure both preventive and reactive.

**Results:**

Model simulations suggest that there is a non-negligible probability (5% to 15%), strongly dependent on reproduction number and probability of developing clinical symptoms, that the escape event is not detected at all. We find that the containment depends on the timely implementation of non-pharmaceutical interventions and contact tracing and it may be effective (>90% probability per event) only for pathogens with moderate transmissibility (reproductive number no larger than R_0_ = 1.5). Containment depends on population density and structure as well, with a probability of giving rise to a global event that is three to five times lower in rural areas.

**Conclusions:**

Results suggest that controllability of escape events is not guaranteed and, given the rapid increase of biosafety laboratories worldwide, this poses a serious threat to human health. Our findings may be relevant to policy makers when designing adequate preparedness plans and may have important implications for determining the location of new biosafety laboratories worldwide.

## Background

The risk associated with the accidental laboratory escape of potential pandemic pathogens is under the magnifying lens of research and policy making communities [1,2]. The recent debate on the genetic manipulation of highly virulent influenza viruses [3,4] has made clear the necessity for quantitative risk/benefit assessment before starting research projects involving biosafety level (BSL) 3 and 4 agents. According to data collected in 2010 and 2011, the number of BSL 4 laboratories worldwide is 38 [5], mostly concentrated in the US (10) and Europe (14). The official number of BSL 3 facilities worldwide is unknown, since most laboratories where research on infectious diseases is carried out and many hospital laboratories operate at safety level 3. Their number, however, is of the order of several thousands: there were 1,362 in the US alone in 2008 [6]. According to data collected in 2010, the number of US workers with approved access to biological select agent and toxin (BSAT) was 10,639 [7]. From 2004 to 2010, 639 release reports were reported to the Centers for Disease Control (CDC), 11 of them reporting laboratory-acquired infections that, however, did not result in fatalities or secondary transmission [7]. A list of recently reported laboratory-acquired infections is available (see [8]). A rigorous risk assessment is a scientific challenge *per se *[9-11]. Although the estimates of the probability of accidental escape are relatively low (0.3% risk of release per lab per year [11]), the increased number of laboratories working on BSL 3 and 4 agents gives rise to estimates projecting an appreciable combined escape risk of potential pandemic pathogens (PPP) in a 10-year window [11]. In addition, for PPP, the relatively small risk of release has to be weighted against the size of the population that could be affected by such an event, the risk of severe or fatal cases and the likelihood of containment before the event could escalate to global proportions. Furthermore, the quantitative analysis of the post-release scenario is complicated by the different social and environmental settings that apply to the more than 1,500 BSL 3 and 4 laboratories around the world [9].

Here, we perform a quantitative analysis of (accidental) post-release scenarios from a BSL facility, focusing on the likelihood of containment of the accidental release event. Although BSL 4 agents, such as Ebola virus and Marburg virus, are considered the most dangerous to handle because of the often fatal outcome of the disease, they are unlikely to generate global risk because of their inefficient mechanism of person-to-person transmission and other features of the natural history of the induced diseases [12,13]. It is therefore understood that the major threat of a pandemic escalation is provided by modified influenza viruses [10], and for this reason we focused our work on the accidental release of novel influenza strain in a densely populated area of Europe. We used a highly detailed agent-based model that specifically considers laboratory workers and their household in order to test the detailed implementation of non-pharmaceutical containment measures in the very early stage of the release/outbreak scenario. The model allowed analysis of the progression of the epidemic at the level of single individual. We could therefore assess the likelihood of containment as a function of a wide range of interventions, and provide a discussion of different geographical settings (for example, rural vs urban seeding) by analyzing the effects of population density and structure. Differently from methods employed to estimate the probability of containing naturally emerging pathogens at the source, here we assumed that epidemiological surveillance is presumably enhanced in areas where BSL laboratories are located, thus increasing the likelihood of quickly detecting symptomatic cases. Moreover, we assumed that this makes it possible to put in place intervention measures (for example, social distancing measures and contact tracing) at the very beginning of the epidemic. A number of factors determine the controllability of an outbreak, including the uncertainty in the efficacy of the containment policies recorded in the literature. For this reason we performed a very extensive sensitivity analysis on the efficacy of implemented policies and the disease natural history. In terms of specific interventions implemented, our analysis is inspired by the experience of an accidental release of severe acute respiratory syndrome (SARS) in August 2003 from a laboratory in Singapore [14]: a total of 8 household contacts, 2 community contacts, 32 hospital contacts, and 42 work contacts were identified, of whom 25 were placed under home quarantine. Both laboratories where the patient had worked were closed as a precautionary measure. Specifically as regards contact tracing, its efficacy for tuberculosis (TB) is ascertained (large-scale studies tracing contacts of TB patients in the US and Canada found high incidence rates of active TB (200 to 2,200 cases per 100,000 individuals) against 5 to 10 per 100,000 in the general population [15-17]). In contrast, contact tracing was performed in the case (described above) of accidental release of SARS and in another case of SARS [18] (1,000 persons traced), but no secondary infections were detected. The two most critical quantities affecting the temporal pattern of spread of influenza viruses, and containment probabilities as well, are the generation time (the distribution of the time interval between infection of a primary case and infection of a secondary case caused by the primary case), and the basic reproduction number R_0_. We analyzed different scenarios by assuming transmissibility comparable to that observed in past influenza pandemics, for example, the 2009 H1N1 virus (namely R_0_ or effective transmissibility in the range 1.2 to 1.6 [19-24]) or 1918 Spanish influenza (R_0_ = 1.8 or higher [25]), and generation time distributions consistent with current estimates for influenza (in the range 2.5 to 4 days [23,26-29]). Beyond these factors, intervention efficacy depends on probability of developing clinical symptoms and length of the incubation period, as they affect, respectively, the probability of detecting cases and the probability of stopping the transmission chain through rapid identification of secondary cases. All these factors make influenza different from other potential pandemic pathogens. For instance, SARS is characterized by a very long incubation period (1 to 2 days for influenza, up to 10 days for SARS [30]) and by a low proportion of infections generated by asymptomatic infections (up to 50% for influenza, negligible for SARS [30]). The R_0_ of SARS was estimated to be slightly larger than that of influenza, namely in the range 2 to 3 [30]. Smallpox, similar to SARS, is another potentially pandemic pathogen characterized by a low proportion of infections generated by asymptomatic infections [30], though characterized by a larger R_0_ (in the range of 5 to 10 [30]). In contrast, Marburg hemorrhagic fever is characterized by a low R_0_ (about 1.5 [12]) and short incubation period (about 2 days, with an overall generation time of 8 to 10 days [12]).

## Methods

**Figure 1:**
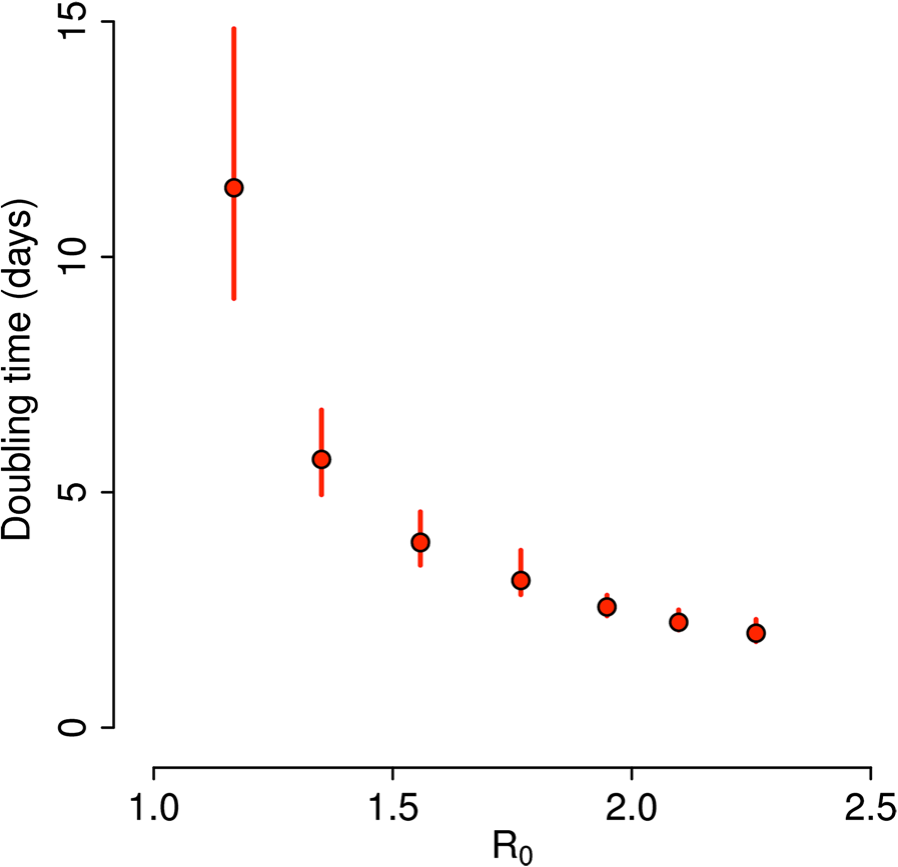
**Doubling time.** Average doubling time (dots) and 95% CI (vertical lines) as a function of R_0_. For each value of R_0_ results were obtained by analyzing 100 uncontrolled (no intervention) simulated epidemics.

In order to provide a quantitative assessment of the containment likelihood and the detailed modeling of interventions we used a stochastic microsimulation model structurally similar to the one used elsewhere (see [19,31]) to generate simulations of pandemic events. The model is a spatially explicit stochastic individual-based model of influenza transmission with force of infection decreasing with the distance and explicit transmission in households, schools and workplaces (see Additional file 1 for details). This model has been validated with data from the H1N1 2009 pandemic [19] and compared and tested against other large-scale computational approaches [32]. The model integrates highly detailed data on country-specific sociodemographic structures (for example, household size and composition, age structure, rates of school attendance, and so on) available from the Statistical Office of the European Commission [33]. These data were used to generate highly detailed synthetic populations. More specifically, census data on frequencies of household size and type, and age of household components by size were used to group individuals into households. Data on rates of employment/inactivity and school attendance by age, structure of educational systems, school and workplace size allowed the assignment of individuals to schools and workplaces or their tagging as inactive, according to their age. Following the available estimates [34-37], the transmission model is parameterized so that 18% of transmission occurs through contacts made at school, 30% within households, 19% in workplaces and 33% in the general community. We made use of state of the art estimates of generation time for influenza viruses in the different settings [38], namely age dependent Weibull distributions (see Additional file 1 for details on the natural history of the virus) with a latent period of 1 day, consistent with estimates of generation time in the range 2.5 to 4 days [23,26-29]. As it is nearly impossible to predict the reproduction number R_0_ of a modified influenza strain (typical values for past influenza pandemics are in the range 1.3 to 2 [23-26,39-44]) we analyzed scenarios with R_0_ varying from 1.1 to 2.5, accounting for the possible larger transmissibility of the modified virus with respect to past influenza viruses. The resulting doubling time of simulations without intervention is shown in Figure 1. We considered containment successful if the disease was eliminated in less than 5 months and resulted in less than 1,000 cumulative cases. The rationale for this choice is that, beyond the obvious requirement of disease elimination, epidemics should be characterized by a relatively low, socially acceptable, cumulative number of cases in a relatively short period of time; otherwise we speak of outbreak. See Additional file 1 for methodological details.

**Figure 2:**
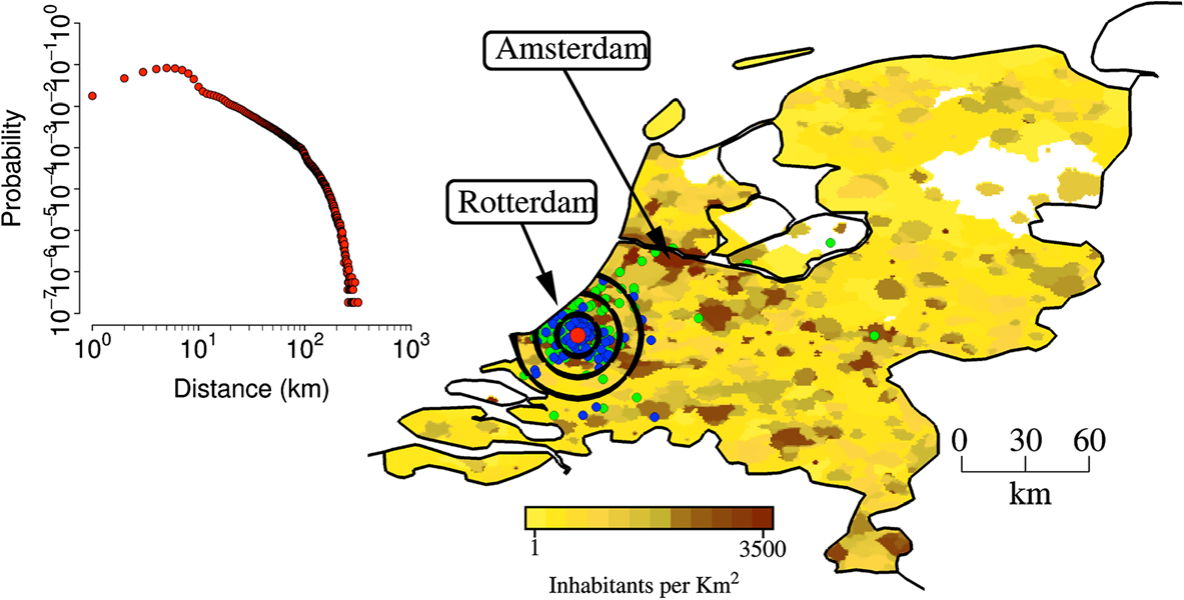
**Study area.** The map shows population density of the Netherlands (colors from yellow to dark brown indicate increasing densities, from 1 to 3,500 inhabitants per km^2^), the location of the laboratory in a randomly chosen simulation (in Rotterdam, red point), the location of the workers houses (blue points), the location of workplaces and schools attended by household members of laboratory workers (green). Black concentric circles indicate distances of 10 km, 20 km, 30 km from the laboratory. The inset shows the probability of commuting to (at) a certain distance by laboratory workers.

Once the initial conditions for the outbreak were set the model generated stochastic ensemble estimates of the unfolding of the epidemic. The infection transmission chain can be analyzed at the level of each single individual and all the microscopic details of the progression of the epidemic in the population can be accessed for each stochastic realization of the escape event. The escape events were identically initialized in a BSL facility in the Netherlands (see Figure 2), by assuming 1 initial infected worker (among 50 to 150 workers; results obtained by assuming a different number of initial infections are analyzed in Additional file 1). This is a fundamental difference of the proposed method with respect to methods employed to estimate the probability of containing naturally emerging pathogens at the source or to analyze the potential effects of bioterrorist attacks: we assume to exactly know the starting point of the outbreak. A second key difference from other studies is the following: we assume that, if ascertained, initial infections generated by the first infected laboratory worker in the network of contacts comprising laboratory colleagues and laboratory workers’ household members may generate an initial warning, and a set of medical/epidemiological analyses are conducted very early to identify the origin of reported symptoms.

We assumed the warning to be issued at the time T_w_ corresponding to the first identification of one of the initial cases. Two key parameters determine the efficacy of subsequent interventions: the first one is probability (P_c_) of identifying initial infections, which is related to the virus specific probability of developing clinical symptoms and the probability of individuals to be actually concerned and report their health status. The second one is the time (T_i_) required to link the initial infections to an accidental release of the modified influenza strain in the laboratory (and not, for instance, to other circulating seasonal influenza viruses) and to activate the containment interventions.

Once the PPP escape event has been detected we considered the following set of containment interventions: (i) isolation of the laboratory, (ii) laboratory workers’ household quarantine, (iii) contact tracing of cases and subsequent household quarantine of identified secondary cases, (iv) school and workplace closure both preventive, on a spatial basis, at the very beginning of the epidemic, and reactive during the entire epidemic.

**Figure 3:**
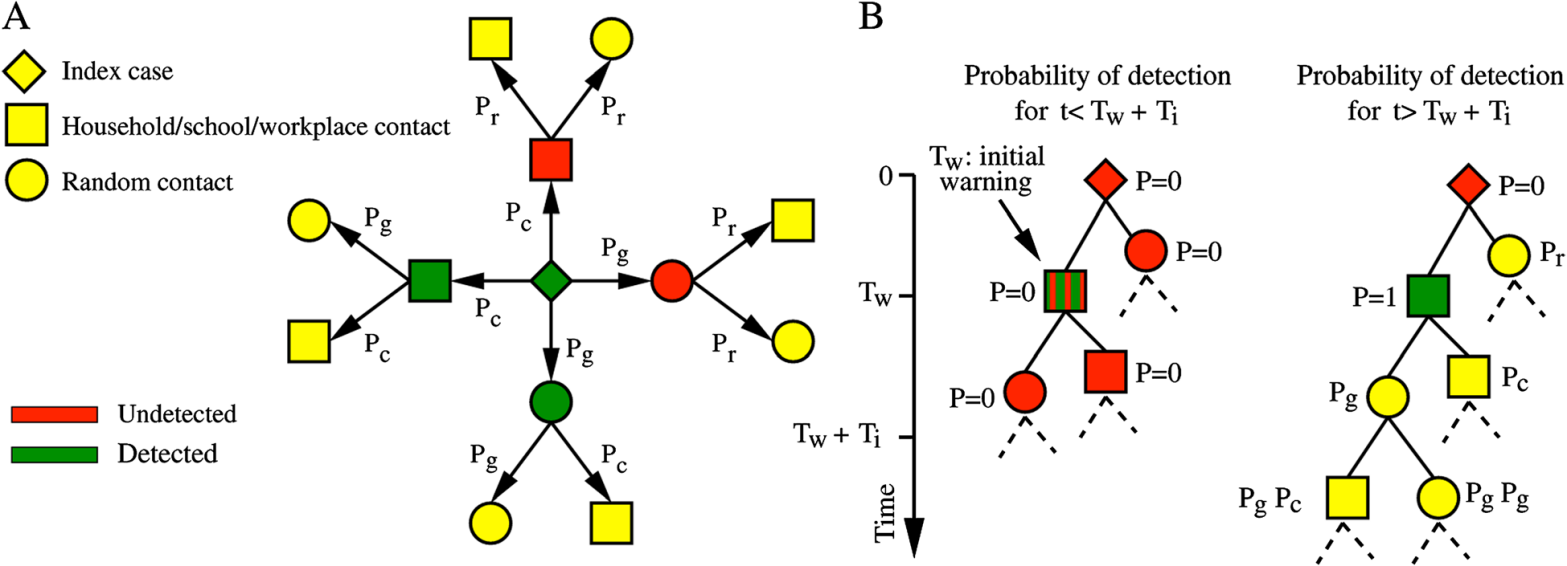
**Contact tracing. (A)** Probabilities of detecting first and second generation cases (the latter conditioned to the detection of first generation cases) triggered by a traced index case. **(B)** Example of network of cases triggered by the initial infected laboratory worker (undetected in this example; the initial warning is triggered by a secondary case in the laboratory), and probability of case detection at time of intervention (T_w_ + T_i_).

For contact tracing, we assumed that once one case is detected, infected close contacts (that is household, school and workplace contacts) of the case are detected with probability P_c_ and can transmit the infection for a certain time (T_t_) before isolation and household quarantine. Cases generated through random contacts in the general population are detected with lower probability (P_g_). We also assume that undetected cases may self-report their health status with a certain probability (P_r_). Parameters characterizing interventions along with reference values and explored ranges are described in Table 1 (see also Additional file 1 for model details). Detailed descriptions of the contact tracing procedure and initial detection of the accidental release are shown in Figure 3A,B respectively. In the following we explore different implementations of the containment interventions and assess their effectiveness by generating stochastic scenario output (SSO) sets, providing for each point in space and time, as given by the resolution of the model, an ensemble of possible epidemic evolutions. We use as a benchmark SSO set the no intervention case, in which the epidemic is assumed to progress without external intervention and a reference SSO set where all the above containment measures are implemented according to the reference value reported in Table 1.

**Table 1:** Model parameters regulating efficacy of interventions

**Variable**	**Description**	**Reference (range)**
P_c_	Infected close contacts detection probability	0.6 (0.4 to 1)
P_g_	Infected random contacts detection probability	P_c_ × 0.5 (0.1 to 1)
P_r_	Infected random contacts self-reporting probability	P_g_ × 0.8 (0.5 to 1)
T_i_	Delay from initial warning to intervention	3 (0 to 30) days
T_t_	Delay from case detection to household quarantine	1 (0 to 4) days
T_p_	Duration of schools and workplaces closure	21 (0, 7, 14, 21, 28) days
D_p_	Radius for schools and workplaces closure	30 (0, 5, 10, 20, 30, 50, 50>) km
F_s_	Fraction of closed schools	0.9 (0 to 0.9)
F_w_	Fraction of closed workplaces	0 (0 to 0.5)

## Results and discussion

Below we discuss the likelihood that the escape of PPP virus will spread into the local population and the ensuing outbreak will be contained by non-pharmaceuticals interventions that are likely the only ones to be available in the early stage of the outbreak.

### Proportion of escape events that will trigger an outbreak

**Figure 4:**
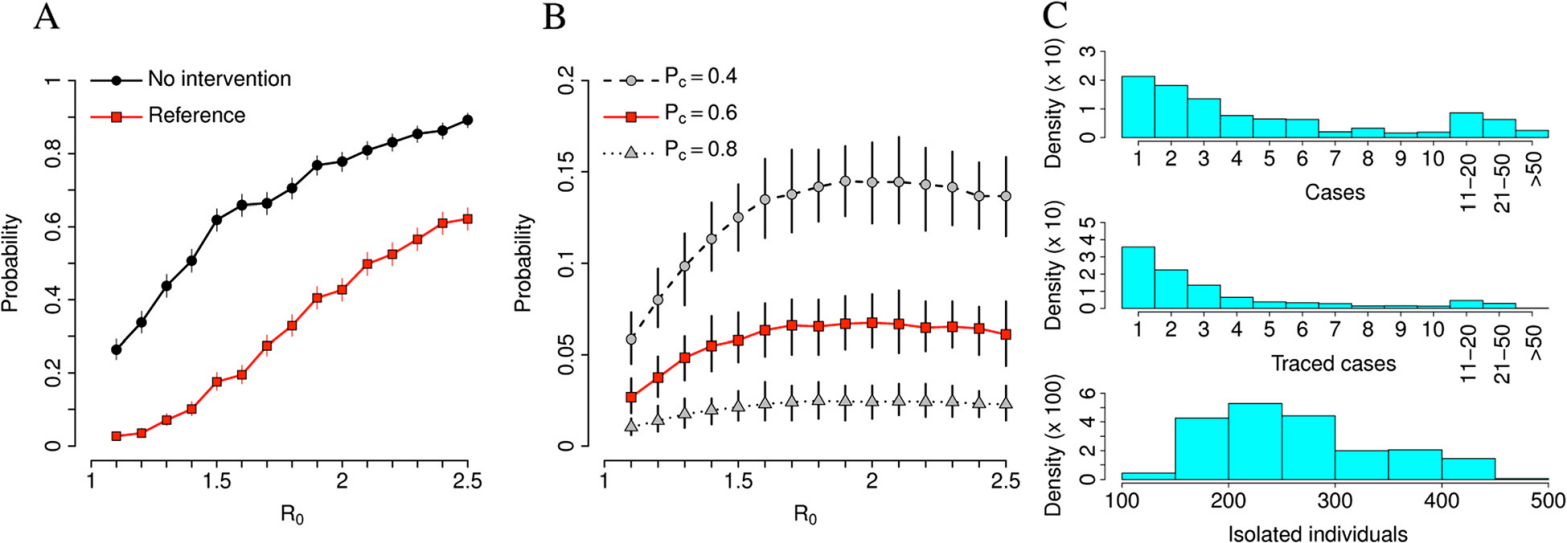
**Reference scenario. (A)** Probability of outbreak for different values of R_0_ by assuming no intervention scenario (uncontrolled epidemics) and reference scenario. **(B)** Probability of undetected epidemics for different values of R_0_ by assuming reference scenario (in red) and reference scenario with different values of P_c_. **(C)** Upper panel: overall number of cases in contained outbreaks by assuming reference scenario and R_0_ = 1.5 (not considering autoextinct epidemics). Middle panel: as upper panel but for the number of traced cases. Lower panel: as upper panel but for the number of isolated individuals (including the laboratory’s contact network). A total of 1,000 simulations were undertaken for each parameter set to produce the results shown.

In order to set a baseline for our investigation it is worth stressing that there is a certain probability that the epidemic goes extinct without any intervention. In general, it is very difficult to estimate this probability, as it depends, beyond other factors, on seeding location (for example, urban vs rural) and contact network of the initial case. In our simulations, all these factors did not vary much as we simulated the initial epidemic seeding to occur always in a BSL facility in a populated area, thus drastically reducing the uncertainty of estimates. The probability of observing an epidemic outbreak in the absence of any interventions (no intervention scenario) is shown in Figure 4A, and it increases from about 25% for R_0_ = 1.1 to values larger than 80% if R_0_ >2.

### Proportion of undetected escape events

Notably, model simulations suggest that there is a non-negligible probability that the escape event is not detected at all. This may happen when no initial cases are detected among laboratory workers and laboratory workers’ household members, but secondary cases are generated through random contacts in the general population. In this case it is reasonable to assume that it is very difficult to ascertain the accidental release of a PPP from the BSL facility and to put in place timely control measures. As shown in Figure 4B, the probability of undetected epidemics increases with R_0_ and it is strongly influenced by the probability of detecting cases. If R_0_ >1.5, it may be as high as 5% when P_c_ = 60% and 15% when P_c_ = 40%. In general, the probability of case detection affects the outcome of intervention options. As we note, to a large extent the detection probability depends on the rate of asymptomatic cases and non-detectable transmissions. In the case of accidental release, the situation is even worse because the probability of detecting cases affects the probability of the timely implementation of the control and containment interventions. As shown in Additional file 1, this probability decreases and eventually vanishes when the number of initial cases is larger than 1.

### Controllability of the escape event

By assuming reference values for the parameters regulating the containment plan, the probability of observing an epidemic outbreak is drastically reduced for all values of R_0_. In particular, containment is likely to succeed for values of R_0_ below 1.5 (probability of outbreak less than 10%, see Figure 4A). The SSO set indicates that for those values of R_0_ the probability of outbreak is largely due to the probability of not detecting the outbreak itself; when the accidental release of the PPP agent is detected in a timely manner, outbreaks are contained with probability close to 100%. The resources required to contain epidemic outbreaks with reference intervention may vary considerably. As shown in Figure 4C, most epidemics are contained at the very beginning, when only few cases are present in the population (median: three infections), thus requiring little effort in terms of contact tracing (median: two traced cases) and overall number of quarantined households. However, it is possible, though not very likely, that containment requires the tracing of several cases (up to 58 traced cases for R_0_ = 1.5, corresponding to the isolation of about 500 individuals). Even more demanding, especially from the social point of view, is the closure of 90% of schools for 21 days in a radius of 30 km around location of initial cases, as assumed by the reference SSO set. The number of cases observed can be easily related to the fatality associated to the outbreak if the case fatality rate (CFR) of the specific PPP agent is known. Unfortunately, the CFR is often not obviously correlated with the transmissibility of the pathogen. In addition, it is extremely difficult to obtain reliable estimates of the CFR during the early stage of an outbreak. A sensitivity analysis of the fatality of the virus can however be performed by applying plausible CFR to the number of cases observed with our approach.

**Figure 5:**
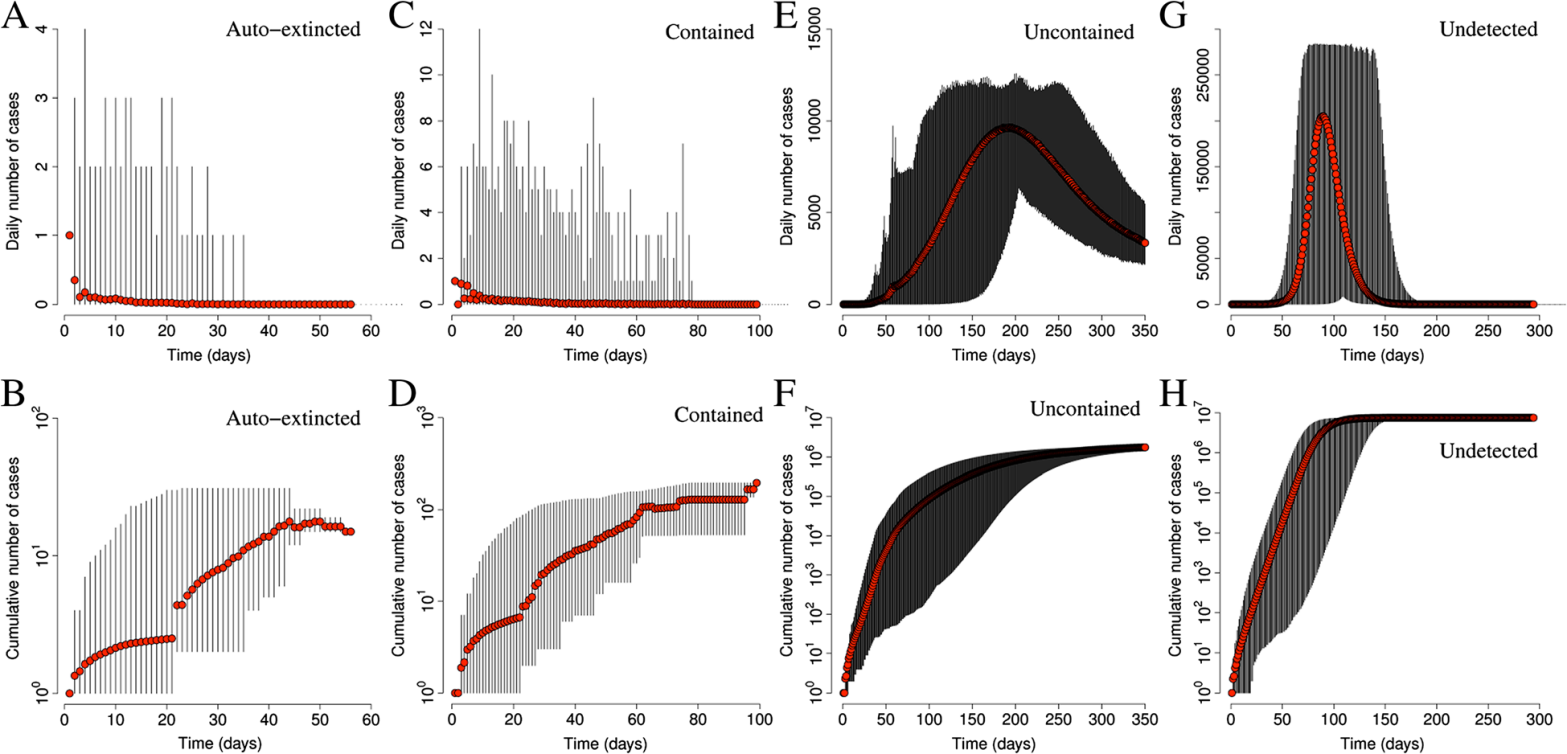
**Epidemic timing. (A)** Average number of daily cases as observed in autoextinct simulated epidemics (red points) with R_0_ = 1.5. Vertical lines represent minimum and maximum daily incidence. **(B)** As in **(A)** but for the average cumulative number of cases. **(C,D)** As **(A)** and **(B)** but for contained epidemics by assuming reference interventions. **(E,F)** As **(A)** and **(B)** but for uncontained epidemics by assuming reference interventions. **(G,H)** As **(A)** and **(B)** but for undetected epidemics. A total of 1,000 simulations were undertaken to produce the results shown.

The timeline of simulated epidemics with R_0_ = 1.5 is shown in Figure 5. Autoextinction occurs in very few days (maximum 57 days) after only few cumulative cases (maximum 10 to 20 cases). A similar pattern is observed for contained epidemics, which may be characterized by a slightly longer duration (maximum 100 days) and slightly larger number of cases (maximum 100 to 200). In both cases, incidence is always less than 20 daily cases. Uncontained epidemics result in long-lasting epidemics (more than 1 year) and produce a large number of cases in a short period of time (larger than 10,000 in 5 months; peak incidence between 10,000 and 15,000 daily cases). Undetected epidemics are shorter (less than 1 year) but are characterized by a much larger number of cases (overall attack rate: 49.5% on average) and peak incidence (between 200,000 and 300,000 daily cases). In addition, these results show the mitigation efficacy of the proposed interventions (specifically household quarantine and reactive school closure on the basis of contact tracing procedures). Moreover, as only 2 different patterns may occur (either the disease quickly dies out after a very limited number of cases or it results in an epidemic outbreak, with many cases in the very first days), these results justify our definition of contained epidemic (disease elimination in less than 5 months and less than 1,000 cumulative cases), though many others are of course equivalent.

### Sensitivity analysis of containment policies

**Figure 6:**
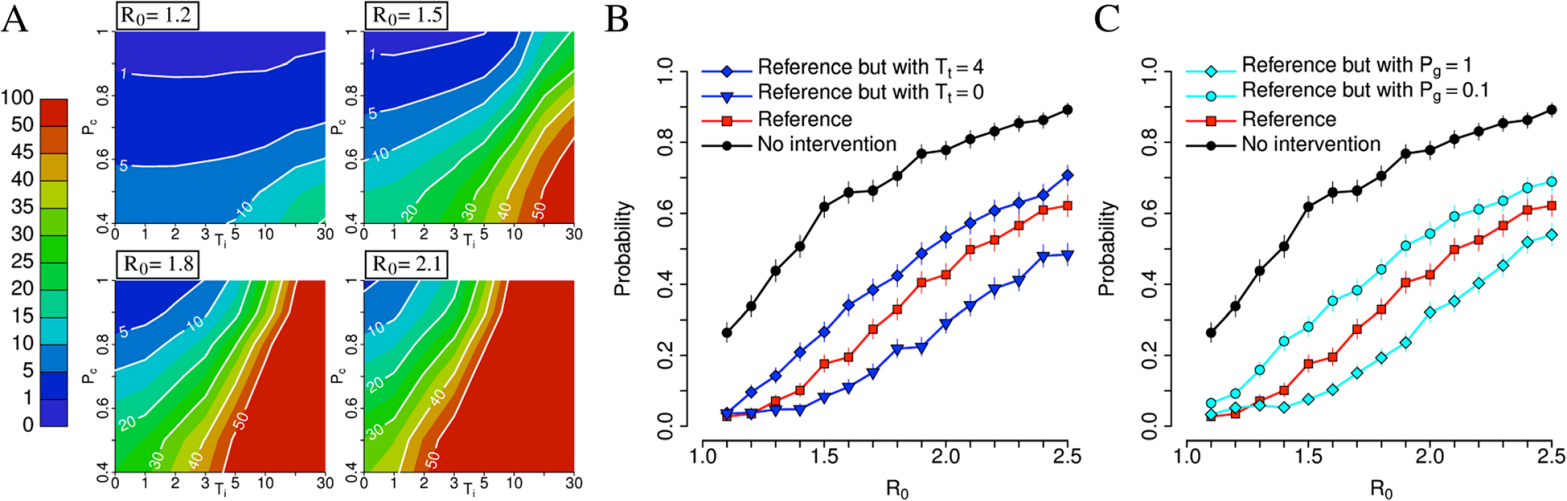
**Sensitivity analysis: contact tracing. (A)** Probability (×100) of outbreak for different values of R_0_ by assuming reference scenario and by varying T_i_ and P_c_. **(B)** Probability of outbreak for different values of R_0_ by assuming no intervention scenario, reference scenario, and reference scenarios with different delays in the isolation of traced cases. **(C)** Probability of outbreak for different values of R_0_ by assuming no intervention scenario, reference scenario, and reference scenarios with different probabilities of identifying cases in the general community. A total of 1,000 simulations were undertaken for each parameter set to produce the results shown.

Results are very sensitive to most of the parameters describing intervention options. By restricting our analysis to parameters regulating contact tracing (thus excluding self-reporting of cases and preventive closure of schools and workplaces) we found that the probability of detecting infections among close contacts of cases and time from initial warning to interventions are the two most critical variables (see Additional file 1 for details). Figure 6A shows sensitivity of results obtained by assuming reference parameters but varying the values of these two parameters. For low values of R_0_ containment is very likely to succeed when P_c_ is larger than 60% (for R_0_ = 1.2) or 80% (for R_0_ = 1.5) even when the delay from initial warning to interventions (T_i_) is much larger than the one assumed by reference simulations (up to 30 or 10 days for R_0_ = 1.2 and 1.5 respectively). For larger values of R_0_, containment is feasible only when P_c_ is larger than 60% and T_i_ is no larger than 3 to 5 days. Figure 6B,C show that other parameters regulating contact tracing can play an important role. In particular, Figure 6B shows that a timely intervention during contact tracing is very critical and Figure 6C shows that it may be important to identify a high number of contacts infected in the general population. This may be difficult in practice but it might be a critical factor for the successful containment. If contact tracing allows the identification of cases in the general community with approximately the same probability of identifying secondary cases in household, school and workplaces, epidemic outbreaks with R_0_ up to 1.6 to 1.7 could be reasonably expected to be contained.

### Effectiveness of preventive school and workplace closure

**Figure 7:**
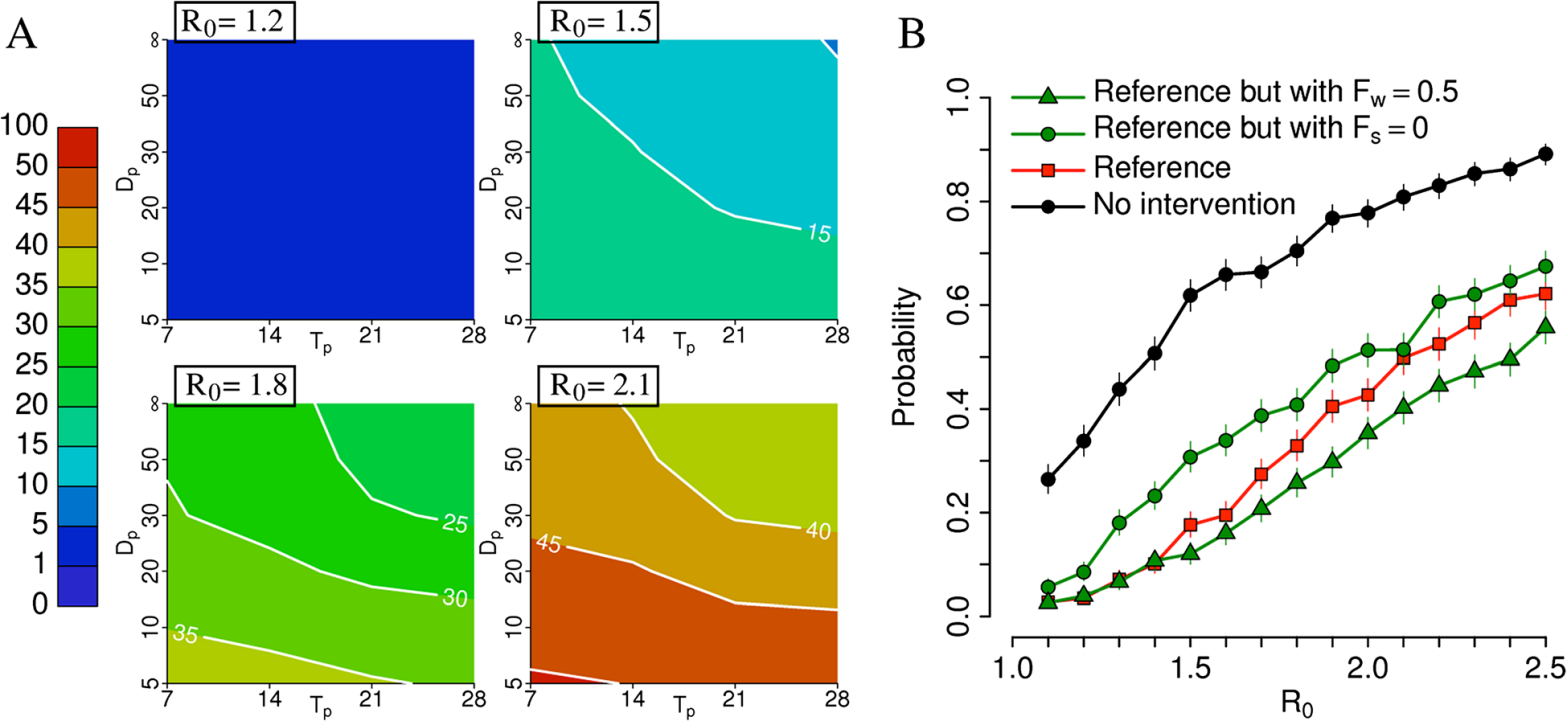
**Sensitivity analysis: school and workplace closure. (A)** Probability (×100) of outbreak for different values of R_0_ by assuming reference scenario with additional workplaces closure (F_w_ = 0.5) and by varying D_p_ and T_p_. **(B)** Probability of outbreak for different values of R_0_ by assuming no intervention scenario, reference scenario, and reference scenarios with different policies regulating school and workplaces closure. A total of 1,000 simulations were undertaken for each parameter set to produce the results shown.

Figure 7B shows that closure of schools (with probability 90%) may be relevant while the additional closure of workplaces (with probability 50%) may be relevant only to decrease the outbreak probability when R_0_ is larger than 1.4. Figure 7A shows that distance for spatial closure of places and duration of closure are irrelevant when R_0_ is 1.2 (as the overall impact of the strategy is not very relevant), while for values of R_0_ = 1.5 or larger, model simulations show that, as expected, the longer the duration and the greater the distance are the lower the probability of outbreak is: duration of 21 days and distance of 30 km represent a good compromise between feasibility and impact. A distance of 30 km for spatially targeted interventions is remarkably larger than that considered in [25] for containing an epidemic in Thailand. This can be explained by looking at the different human mobility patterns in Thailand, where most of commuting is within 5 km, and the Netherlands, where commutes of 10 to 30 km to go to work or school are common [45] (see inset of Figure 2 and Additional file 1 for details).

### Geographical context analysis

**Figure 8:**
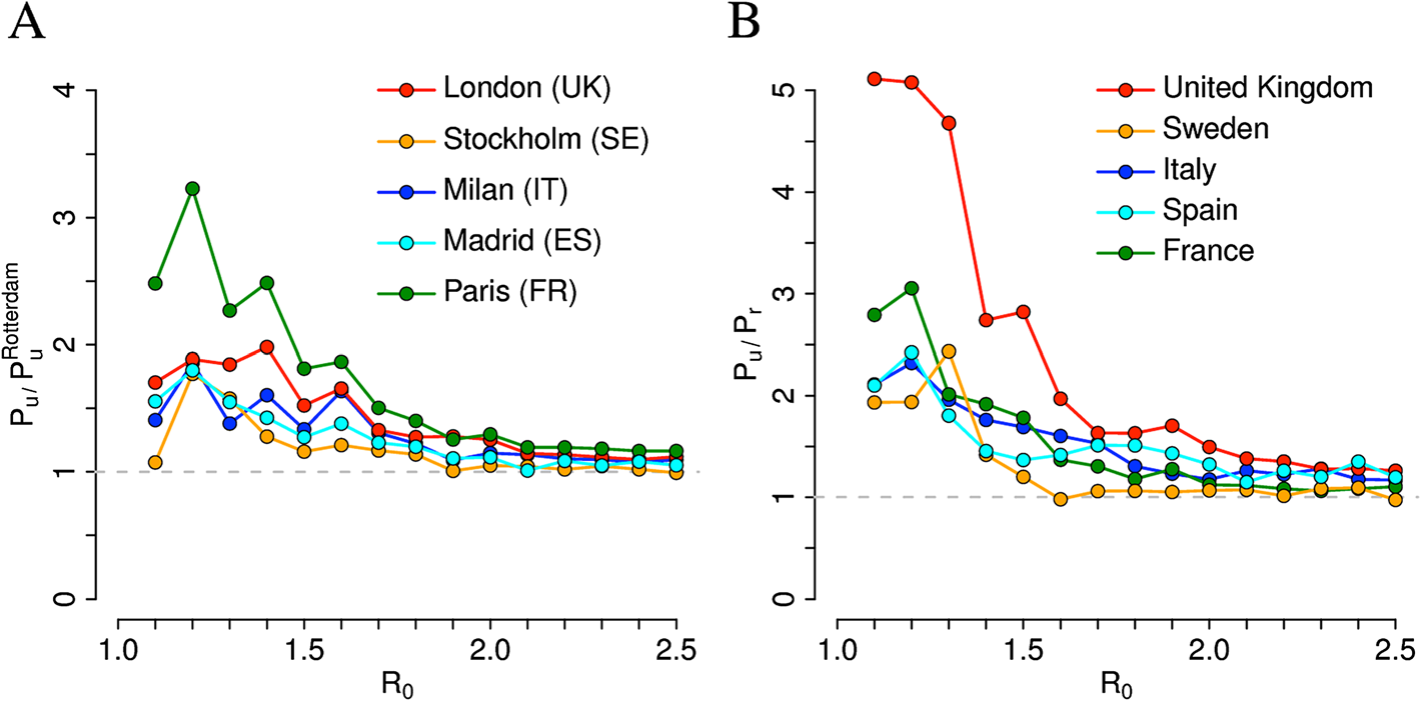
**Geographical variability. (A)** Ratio between probability of outbreak in different urban areas and probability of outbreak in Rotterdam (The Netherlands) for different values of R_0_ by assuming reference scenario. **(B)** Ratio between probability of outbreak in urban and rural areas in different countries for different values of R_0_ by assuming reference scenario. Urban areas as in **(A)**; rural areas are low population density areas in Wales (UK, 80 km north of Cardiff), Uppland (SE, 100 km north of Uppsala), Sardinia island (IT, 50 km east of Sassari), Andalusia - Castile la Mancha (ES, 50 km northeast of Cordoba), Centre-Burgundy (France, 80 km southeast of Orleans). Note that the reported value of R_0_ refers to that of simulations carried out for Rotterdam (The Netherlands); comparative results for other countries are obtained by assuming the same transmission rates in the different social contexts (that is the same probability of infection transmission given a contact in a certain setting) as in Rotterdam. A total of 1,000 simulations were undertaken for each parameter set to produce the results shown.

The probability of containing an epidemic outbreak may also depend on the BSL laboratory location and the sociodemographic structure of the population. This is shown in Figure 8A where we compare results obtained for Rotterdam (The Netherlands) with those obtained by simulating the epidemic spread emerging from BSL facilities in other urban areas of Europe. We found that Rotterdam likely represents the best case scenario among those analyzed in this paper: for instance, the probability of observing an epidemic outbreak in Paris, by assuming reference interventions, may be 200% to 300% larger than that estimated for the Netherlands if R_0_ <1.5. Differences reduce drastically for larger values of R_0_. Without considering control measures, the probability of observing an epidemic outbreak after virus escape is quite similar to that in the Dutch scenario: slight differences can be observed for low values of R_0_. Such large differences may be due to dissimilarities in sociodemographic characteristics of French and Dutch populations because, despite a general similarity, some marked country-specific features such as age structure and average household size exist. However, although quantitatively different, the general patterns obtained by varying P_c_ and T_i_ are the same observed in the Dutch case. Detailed results for Paris are discussed in Additional file 1. We also found that the probability of observing an epidemic outbreak when the BSL laboratory is located in a rural region is systematically lower than that estimated for urban areas (see Figure 8B). For instance, given a BSL facility located in the UK, we found that the probability of epidemic outbreak when the pathogen is accidentally released from a hypothetical BSL laboratory in Wales (UK) may be three to five times lower than that estimated for a BSL laboratory in London if R_0_ <1.5. These differences are ascribable to differences in population density and sociodemographic structure, as discussed in [31]. These results are discussed in detail in Additional file 1.

### Impact of additional intervention

We found that results are not very sensitive to the probability of self-reporting (P_r_) and to the initial set of interventions on the initial network of contacts comprising laboratory workers and laboratory workers’ household members. The reference scenario assumes the closure of the laboratory and the quarantine of the households of laboratory workers. We explored the possibility of extending these interventions and to preventively close all workplaces and schools attended by relatives of laboratory workers. We found that closing the laboratory is the only intervention leading to a certain reduction of the outbreak probability. Additional interventions are of little impact. We report on these findings in Additional file 1.

## Conclusions

Our results suggest that containment is likely to succeed by employing social distancing measures only if R_0_ is no larger than 1.5. Containment could be feasible even for larger values of R_0_ in cases of very timely intervention both in recognizing the accidental release and during contact tracing and high probability of detecting secondary cases in the same household, school or workplace as a newly identified case. Overall, these results suggest that success in containing an accidentally released potentially pandemic influenza virus by employing social distancing measures only is uncertain: containment probability for a virus with transmissibility comparable to many of the estimates for the 2009 H1N1 virus (namely R_0_ or effective transmissibility in the range 1.2 to 1.6 [19-24]) is reassuring, even though containment is not guaranteed. Should the transmissibility of the pathogen be comparable to that of the 1918 Spanish influenza (R_0_ = 1.8 or higher [25]), containment success would be seriously compromised. A further relevant finding is the strong impact of the BSL laboratory location. Rural areas have a fivefold increase in containment probability with respect to densely populated urban areas. Similarly, we observe differences according to the sociodemographic structure of the geographical region. These results provide data with potential use in defining policies for deciding the most appropriate location of BSL laboratories.

Our simulations do not account for the possible use of pharmaceutical interventions. While the availability of an effective vaccine is highly questionable in case of accidental release of genetically manipulated influenza viruses from BSL facilities, the use of antivirals at the very beginning of the epidemic is an option that could be considered. If used for treatment of cases and prophylaxis of close contacts (for example, household and school contacts) only, however, the benefit should not be very different from that obtained by assuming household quarantine and reactive school closure, as this paper does. Moreover, it requires a timely administration (within 2 days from symptoms onset [25,46-50]) to be effective. Geographical targeting of a large fraction of the population is a completely different option that could be considered: on the one hand it could lead to drastically increasing the probability of containment but on the other hand also poses serious logistical challenges [25,47].

The preventive immunization of laboratory workers (see for instance the Special Immunization Program in the US [51]) is another option not considered in this work. Although for diseases for which a vaccine is available this is a measure to take into account (for instance, the incidence of hepatitis B virus (HBV) infection among laboratory workers in the UK has significantly dropped because of the availability of immunization [52]), this measure is highly questionable for genetically modified influenza viruses, not to speak of influenza viruses for which no vaccine is currently available, for example, A(H7N9).

In summary, our results suggest that public health authorities should be prepared to put in place a set of social distancing interventions, for example, contact tracing and closure of schools and workplaces on a geographical basis. Moreover, as it is nearly impossible to get accurate estimates of R_0_ (as well as case fatality rate) for a new virus at the very beginning of the outbreak, in order to maximally reduce the risk of a global pandemic the possibility of timely targeting a large fraction of the population with antivirals (as a prophylactic measure on a geographical basis) or establishing quarantine areas should not be set aside, even though this calls for the development of detailed intervention plans and requires public health agencies to put in place containment efforts hardly achievable in most places in the world. Where the pandemic pathogens are concerned, short generation time and asymptomaticity are among the most critical factors that make accidental release of influenza viruses difficult to contain.

Qualitatively, the results do not vary much by considering different seeding locations. However, containment probabilities are affected by several factors, including population density and sociodemographic structure. These findings may have an important impact on policies: our results strongly suggest the location of new BSL facilities worldwide should be carefully chosen, for instance with priority given to rural areas and, when this is not feasible, by taking into account density and structure of the population in urban areas. This may make the difference, especially for pathogens with low to moderate transmissibility. Of course, these decisions should also be based on other factors not considered in this study, for example, population vulnerability to infectious agents, risk factors, structure of the health system, possibility of putting in place a rapid response program. Simulated scenarios emerging from detailed models such as the one presented here may inform quantitatively the process of identifying locations that minimize risk. Finally, it is worth remarking that the presented approach can be generally extended to other pathogens that can be classified as dual use research of concern if we have the appropriate information on the pathogens, mechanism of transmission and natural history of the disease.

## Competing interests

The authors declare they have no competing interests.

## Authors’ contributions

SM and AV conceived of the study. SM, MA and LF performed the experiments. SM, MA, LF and AV analyzed results and wrote the manuscript. All authors read and approved the final manuscript.

## Pre-publication history

The pre-publication history for this paper can be accessed here:

http://www.biomedcentral.com/1741-7015/11/252/prepub

## Supplementary Material

Additional file 1 **Supporting material.** Model details, additional results. Click here for file
