# Supplementary material for: Containing the accidental laboratory escape of potential pandemic influenza viruses
Source: BMC Med. 2013 Nov 28;11:252. doi: 10.1186/1741-7015-11-252 (PMC4220800; doi:10.1186/1741-7015-11-252)
Supplement: Additional file 1 — Supporting material. Model details, additional results. [file 1741-7015-11-252-S1.pdf]

Containing the accidental laboratory escape of potential pandemic influenza viruses

Stefano Merler[1], Marco Ajelli[1], Laura Fumanelli[1,2], Alessandro Vespignani[3,4,5]

[1] Bruno Kessler Foundation, Trento, Italy
[2] Department of Mathematics, University of Trento, Trento, Italy
[3] Laboratory for the Modeling of Biological and Socio-technical Systems, Northeastern University, Boston, MA
[4] Institute for Quantitative Social Sciences at Harvard University, Cambridge MA
[5] Institute for Scientific Interchange Foundation, Turin, Italy

Supplementary Material

Contents

1 Materials and methods **2**
1.1 Sociodemographic model 2
1.2 Human mobility 2
1.3 Disease transmission model 2
1.4 Natural history of influenza and model calibration 4
1.5 Seeding of the accidental escape 6
1.6 Control measures and self reporting 6

2 Additional results **8**
2.1 Full sensitivity analysis on contact tracing 8
2.2 Sensitivity analysis on parameters regulating intervention measures 8
2.3 Different number of index cases 11
2.4 Different seeding locations 11

1 Materials and methods

1.1 Sociodemographic model

We performed our analysis by using an individual-based stochastic simulation model previously developed for Europe and employed in [1] to derive synthetic contact matrices for 26 countries, which is a refined version of a model already used to investigate the spread of the 2009 H1N1 influenza pandemic in Europe [2, 3]. Highly detailed country-specific socio-demographic data on age structure, household size and composition, employment rates by age, educational system were used to generate a synthetic population where each individual is characterized by an age, a household membership and an occupation between student, worker and inactive (unemployed/retired).

Data on households and employment rates are provided by the Statistical Office of the European Commission (Eurostat, `http://epp.eurostat.ec.europa.eu`); data on educational organization are taken from the Education, Audiovisual and Culture Executive Agency of the European Commission (`http://eacea.ec.europa.eu`) and from the UNESCO Institute for Statistics (`http://www.uis.unesco.org/Pages/default.aspx`).

Briefly, households are built by using a heuristic model matching marginal distributions of household size, age of household members by size (and thus the age structure of the total population), composition (single adult or couple, with or without children), as derived from available census data, and maintaining realistic generational age gaps between household members. Then, an occupation is assigned to individuals: every member of the population either goes to school or workplace, or remains at home (for instance as a retired or family worker), following census data on employment rates by age. Students are assigned to the correct school level of the educational system according to their age. Real average distributions of school and workplace size are matched in the model.

Full details on the initialization of the population can be found in [1].

1.2 Human mobility

As mentioned in Section 1.1 in our model each household, school and workplace has a specific geographic location. In order to assign students and workers to a school or workplace, we use recent estimates on the commuting pattern of individuals in industrialized countries. Specifically, as proposed in [4] and used for modeling influenza transmission in [2, 3], daily internal commuting between home and school/workplace is modeled in such a way that probability density function of travel distances d complies with a truncated power–law distribution:

$$f(d) \quad = \quad (d + r_g^0)^{-\beta_r} \exp\left\{-\frac{d}{\kappa}\right\}, \tag{1}$$

where $r_g^0 = 5.8$km, $\beta_r = 1.65$ and $\kappa = 350$km [2, 3, 4].

Full details on mobility patterns considered in the model can be found in [2].

1.3 Disease transmission model

Influenza transmission was simulated by a stochastic spatially structured individual–based model. Each individual, explicitly represented in the model as a simulated agent, is characterized by household membership, school/workplace membership (if any), age and epidemiological status.

Each household, school and workplace lies in a specific geographic location. Given the short time period of interest here -of the order of weeks-, once the population is initialized, it is kept constant through the course of the simulation.

The adopted epidemic flow follows the classic Susceptible-Latent-Infectious-Removed scheme: a susceptible individual can become latent after a contact with an infectious individual; after a given period of time -the latent period- latent individuals (i.e., individuals who got infected but that are not able to transmit the disease yet) become infectious; after a given period of time -the infectivity period- infectious individuals recover and they become immune to the disease.

Specifically, as in [2, 3, 5, 6, 7], at any time t of the simulation (time step $\Delta t = 1$ day), any infectious individual i has a probability

$$p_{i,j,\rho}(t) \quad = \quad 1 - e^{-\Delta t \cdot \lambda_{i,\rho}(t)}$$

of infecting a susceptible individual j between her/his household members ($\rho = H$), schoolmates (if any, $\rho = S$), work colleagues (if any, $\rho = W$) and in the general community ($\rho = G$).

In particular, transmission in household is modeled as:

$$\lambda_{i,H}(t) \quad = \quad \frac{\beta_h k_{h,a}(t - \tau_i)}{h_i}$$

where

- h_i is the number of individuals living in the same household of individual i;
- β_h is the transmission rate in households;
- $k_{h,a}(\sigma)$ is the infectivity of individual i, aged a years, in household, σ days since infection (see Section 1.4 for details on function k);
- τ_i is the time at which individual i was infected.

If individual i is a student, transmission in school is modeled as:

$$\lambda_{i,S}(t) \quad = \quad \frac{\beta_s k_{s,a}(t - \tau_i)}{s_i}$$

where

- s_i is the number of individuals attending the same school of individual i;
- β_s is the transmission rate in schools;
- $k_{s,a}(\sigma)$ is the infectivity of individual i, aged a years, in school, σ days since infection;
- τ_i is the time at which individual i was infected.

If individual i is a worker, transmission in workplace is modeled as:

$$\lambda_{i,W}(t) \quad = \quad \frac{\beta_s k_{w,a}(t - \tau_i)}{w_i}$$

where

- w_i is the number of individuals working in the same workplace of individual i;
- β_w is the transmission rate in workplaces;
- $k_{s,a}(\sigma)$ is the infectivity of individual i, aged a years, in workplace, σ days since infection;
- τ_i is the time at which individual i was infected.

Transmission in the general community is modeled as:

$$\lambda_{i,G}(t) = \frac{\beta_g k_{g,a}(t-\tau_i) f(d_{i,j})}{\sum_{j=1}^{N} f(d_{i,j})}$$

where

- N is the total number of individuals in the population;
- β_g is the transmission rate in the general community;
- $k_{g,a}(\sigma)$ is the infectivity of individual i, aged a years, in the general community, σ days since infection;
- τ_i is the time at which individual i was infected.
- $d_{i,j}$ is the geographical distance between individual i and j;
- f is the kernel function reported in Equation (1), which modules the transmission in the general community on the basis of the commuting pattern of individuals of the population as detailed in [2, 3, 4].

1.4 Natural history of influenza and model calibration

Model calibration was made on the basis of empirical data on both past influenza epidemics and the 2009 H1N1pdm pandemic. Following the state-of-the-art estimates on the generation time distributions and infectiousness profiles for the 2009 H1N1 influenza pandemic given in [8], we consider infectiousness over time to depend on the context where interaction occurs and on the age of the case - the population is categorized as "children", i.e., individuals aged less than 10 years, and "adults", i.e., all other individuals in the population. Specifically, infectiousness profiles $k_{\rho,a}(t-\tau_i)$ (where i, ρ, a, t and τ_i are defined as in Section 1.3) are derived from discretized Weibull distributions with an offset of 1 (which is equivalent to having a latency period of 1 day). This leads to setting-specific values of the generation time which are: 3.7 days (standard deviation: 3.1 days) for children in household; 1.1 days (standard deviation: 0.4 days) for children at school and in the community; 2.3 days (standard deviation: 2.9 days) for adults. The distributions of the generation times are reported in Figure S1A.

The model has only four free parameters, namely the transmission rates in the different social contexts. Setting-specific transmission rates were estimated in such a way that the proportions of cases in the different settings are consistent with empirical estimates [6, 9, 10, 11], namely 30% of cases in households, 18% in schools, 19% in workplaces and 33% in the general community. As in [3, 5], different transmissibility scenarios can be obtained by multiplying all transmission

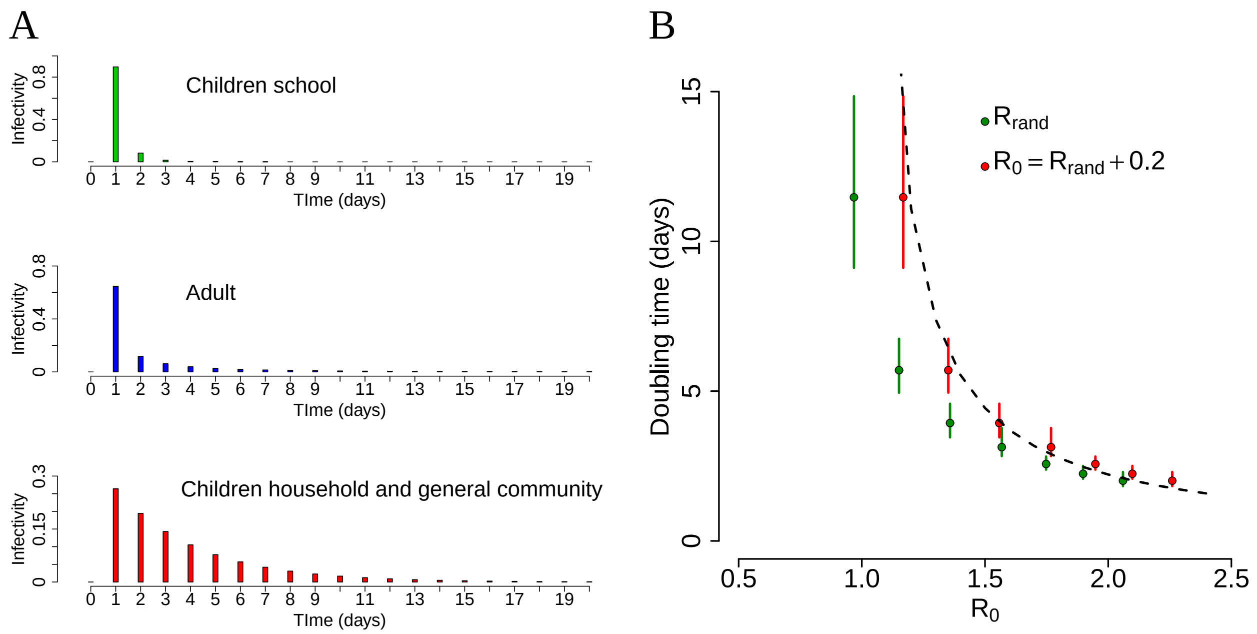

Figure S1: **A** Probability distribution of context and age specific generation time. **B** Epidemic doubling time as a function of R_{rand} and R_0, as resulting from Equation (2). The dashed line represents the plot of Equation (3), where $T_g = T_{\text{eff}} = 3.2$ days.

rates for suitable constant values. Choosing generation time distributions and transmission rates determines the value of the epidemic doubling time -i.e., the time required for the number of infectious individuals to double-, which is a key quantity for the assessment of the effectiveness of containment strategies.

The basic reproduction number R_0 is defined as the average number of secondary infections caused by a typical primary infection in a fully susceptible population [12]. However, in complex models with highly heterogeneous mixing, as the one used in this work, the definition of a "typical" infectious individual is unclear. Therefore, in the parametrization procedure we used R_{rand}, in place of R_0, which is defined as the average number of secondary infections generated by a randomly selected individual in a fully susceptible population [6]. Ferguson et al. [6] found that for US and UK populations, the following relation holds:

$$R_0 \quad = \quad R_{\text{rand}} + 0.2 \qquad (2)$$

In [6] it has been found that this relation depends on the demography of the considered country; in fact the adjustment term 0.2 (which was necessary for US and UK but not for Thailand [5, 6]) was motivated by a different level of household transmission in the US and UK models, due to smaller household sizes and higher transmission levels in schools/workplaces, with respect to the Thai model. As the demography of the Netherlands (and of France) is closely related to that of the UK [1], we use the same adjustment term 0.2.

In our simulations having transmission parameters leading to $R_{\text{rand}} = 1.5$ we estimate an epidemic doubling time of 3.13 days, which is in excellent agreement with the estimates given in [6] (namely 3.16 days) for the simulation having $R_{\text{rand}} = 1.5$ and $R_0 = 1.7$. This reinforces the choice of using Equation (2) for defining R_0 (see Figure S1B). In addition, this allows a simpler comparison between our results and the ones reported in the literature: thus all results are presented in terms of R_0 (instead of R_{rand}).

For classic homogeneous mixing SIR models, it is well known [6, 13] that $R_0 = 1 + rT_g$, where T_g is the average generation time and r is the exponential growth rate of the epidemic. Moreover, the doubling time T_d can be written in terms of r as $T_d = \ln(2)/r$ and thus, for a classic SIR model, we obtain

$$T_d = \frac{\ln(2)T_g}{R_0 - 1} \tag{3}$$

As we are considering setting and age specific generation time distributions, we do not know the value of T_g to be used in Equation (3). However, we want to test whether this approximation still holds for our highly heterogeneous model and, if so, for which value of the generation time (that we call T_{eff}). By minimizing the quantity

$$\sum_{j=R_0\in\{1.1,\ldots,2.3\}} \left[\frac{Y_j - y_j(T_{\text{eff}})}{Y_j}\right]^2$$

where $y_j = \ln(2)T_{\text{eff}}/(j-1)$ and Y_j is the doubling time of the simulations, we find $T_{\text{eff}} = 3.2$ days. This result is in perfect agreement with the findings reported in [6] and, moreover, this relation represents a good approximation in the explored range $R_0 = 1.1 - 2.3$ (see Figure S1B). This justifies the choice (already used for instance in [3, 5]) of multiplying the transmission rate for suitable scaling factors in order to obtain different transmissibility scenarios.

1.5 Seeding of the accidental escape

We initialize the epidemic with one single case in a laboratory located in Rotterdam, the Netherlands. For scenarios with a different number of initial infectives and other seeding areas (namely, urban and rural France) see Sections 2.3 and 2.4, respectively. In every simulation the specific position of the laboratory is assigned by randomly choosing a cell of Rotterdam; the facility comprises 50-150 workers. Since the first case is a laboratory worker, the uncertainty on the initial evolution of the epidemic is greatly reduced for instance with respect to the case of a natural emergence of a new pandemic strain (see for instance [5, 14]): in fact, in the case of an accidental escape from a BSL facility, the index case is likely to be an adult worker whose workplace has well determined features (size and geographic location).

1.6 Control measures and self reporting

We consider various measures for controlling the epidemic spread after the escape from the laboratory:

- Laboratory isolation: as a first intervention implemented T_i days after the ascertainment of the pathogen escape event (occurring at time T_w), the BSL facility is closed for 20 days (which is the maximum allowed time for the duration of viral shedding of an infectious individual, see Figure S1).
- Quarantine of laboratory workers' households: in addition to BSL facility closure, T_t days after case detection, laboratory workers and members of their households are quarantined for 20 days.

- Contact tracing: contacts of a case are identified and traced in the population; among these, detection of secondary cases occurs with a certain probability (P_c if they are close contacts of the primary infector, P_g if they have been infected through random contacts in the general community), and ensuing isolation and household quarantine are put in place, T_t days after, for 20 days.

- School/workplace closure: a given percentage of schools F_s and workplaces F_w is closed for a period T_p either preventively (as soon as the escape event is identified and within a certain distance D_p from the laboratory) or reactively (after the detection of a case involved in contact tracing or the self-reporting of health status by an undetected case in the school/workplace).

In addition, the effect of self-reporting of undetected cases, occurring with probability P_r, has been evaluated.

Description, reference values and explored ranges for the parameters regulating control measures (and self-reporting) are listed in the following (see also Figure 1 in the Main Text).

- P_c is the probability of detecting an infected close contact (i.e., a household or school/workplace contact) of a case. This probability accounts for the combination of two factors: the probability of developing symptoms (which is clearly a characteristic of the pathogen) and the probability of detecting a case, given that she/he is symptomatic. The probability of being symptomatic for past influenza viruses has been estimated to be in the range 50%–75% [5, 14, 15], but it may be even higher for a new, more aggressive, strain. In addition, it is expected that, in case of an emerging pandemic, healthcare systems would be able to identify around 90% of symptomatic cases [5, 6, 7]. Therefore, the combined close contact detection probability is assumed to be in the range 40%–100%. As a reference value we assume $P_c = 0.6$.

- P_g is the probability of detecting an infected "random" contact of a case, where random contact means a contact occurred in the general community (and thus excluding household and school/workplace contacts). Given the greater difficulty in identifying such contacts, we assume P_g to be a fraction ranging from 10% to 100% of P_c; the reference value we used is $P_g = 0.5P_c$ (and thus $P_g = 0.3$).

- P_r is the self-reporting probability of undetected infected individuals. This probability is assumed to be 50%, 80% (reference value) or 100% of P_g; thus the reference value results to be $P_r = 0.24$.

- T_i is the delay from the initial warning to the start of control strategies. In the case of an accidental virus escape from a BSL facility, public health systems would be able to readily react to the emergency after the first case infected by the new virus strain has been detected. However, it is hard to estimate a reliable range for this delay; therefore we decide to explore a wide range of possible values, namely 0–30 days (with a reference value of 3 days).

- T_t is the time necessary to put in place household quarantine after case detection. Since, differently from measures such as antiviral prophylaxis, household quarantine does not involve any pharmaceutical treatment, it can be put in place readily after case detection. The explored range considered is 0–4 days (reference value: 1 day) [5].

- T_p is the duration of school/workplace closure in case of adoption of this measure; this may last up to several weeks, although even a closure period of 3 weeks for schools has been shown to have an impact in controlling the epidemic [6]. We thus consider a reference value of 21 days and explore also the cases of 0, 7, 14 and 28 days.

- D_p is the distance from the laboratory within which preventive measures of school/workplace closure are implemented. Given the average commuting distance traveled by workers in the Netherlands (as predicted by our model [2] and also confirmed by other studies [16]) to be 8.9 Km (95%CI: 1.6-19.2 Km), we assume a reference value for D_p of 30Km. We further explore other values, namely 0, 5, 10, 20, 50Km and the whole country.

- F_s is the fraction of schools closed for the period T_p. As reference value we assume $F_s = 0.9$. In addition, we explore strategies not involving school closure and thus $F_s = 0$.

- F_w is the fraction of workplaces closed for the period T_p. As, even for a relatively short period of time, workplace closure requires a bigger social effort than school closure, as reference value we assume that workplaces are not involved in the closure policy (and thus $F_w = 0$). However, we further investigate the case where 50% of workplaces get closed for a period T_p.

2 Additional results

2.1 Full sensitivity analysis on contact tracing

Main parameters regulating contact tracing strategy are P_c, P_g, T_i, T_t and T_p. In this analysis, we keep $P_r = 0$ and $F_w = 0$ fixed, we consider school closure to be only reactive (with $F_s = 0.9$), and let the other parameters vary. The effect of the different parameters in shaping outbreak probability can be explained by a linear model having P_c, P_g, $\log(T_i)$, T_t and T_p as independent variables. The goodness of fit is highlighted by the high values of the coefficient of determination R^2, which is estimated to be in the range 0.88–0.95 (see Table S1 and Figre S2A). For each choice of R_0, all these variables are strongly significant, except for T_p which is significant only for $R_0 < 1.5$ (see Table S1 and Figure S2B) and thus we conclude that reactive school closure is important for containment only for mild epidemics. In addition, this analysis highlights that the two most relevant parameters are P_c and T_i, i.e., the larger the fraction of detected cases and the lower the delay in starting interventions, the lower the probability that a virus escape leads to an epidemic outbreak.

2.2 Sensitivity analysis on parameters regulating intervention measures

We aim at estimating the impact of different assumptions on the value of the parameters regulating intervention on outbreak probabilities. This analysis is performed by changing one-by-one parameter values and results are shown in Figure S3.

One of the most important parameters is P_c. Figure S3A shows that for $P_c \geq 0.8$ outbreak probability for $R_0 \leq 1.5$ is always below 5%: this is due to the fact that only a negligible fraction

R_0	Intercept	T_i	P_c	T_t	P_g	T_p(p-value)	R^2
1.1	0.1341	0.0651	-0.14	0.0033	-0.0263	-5e-04 (0.000713)	0.939
1.2	0.1883	0.0959	-0.1947	0.005	-0.0353	-6e-04 (0.001597)	0.948
1.3	0.23	0.1226	-0.2327	0.0066	-0.038	-4e-04 (0.045802)	0.951
1.4	0.2697	0.1449	-0.2625	0.0078	-0.044	-6e-04 (0.033768)	0.947
1.5	0.3037	0.1636	-0.2886	0.0086	-0.0437	-5e-04 (0.144265)	0.944
1.6	0.341	0.1782	-0.3131	0.0094	-0.046	-7e-04 (0.032911)	0.943
1.7	0.3613	0.1926	-0.3217	0.0101	-0.0468	-4e-04 (0.286948)	0.937
1.8	0.3856	0.2033	-0.3338	0.0107	-0.0467	-4e-04 (0.341388)	0.932
1.9	0.4072	0.2118	-0.3374	0.0107	-0.046	-6e-04 (0.225857)	0.925
2	0.4262	0.2183	-0.3406	0.0112	-0.048	-4e-04 (0.378901)	0.917
2.1	0.4498	0.2239	-0.3542	0.0116	-0.0455	-2e-04 (0.676714)	0.912
2.2	0.4625	0.2291	-0.355	0.0119	-0.0443	-2e-04 (0.707177)	0.905
2.3	0.4757	0.2334	-0.3524	0.0119	-0.0455	-2e-04 (0.779472)	0.899
2.4	0.4928	0.2354	-0.3544	0.0119	-0.044	-3e-04 (0.661867)	0.888
2.5	0.5055	0.2379	-0.3522	0.0122	-0.0465	-3e-04 (0.619435)	0.884

Table S1: R_0, coefficients of the linear model between outbreak probability and all parameters regulating the interventions and coefficient of determination R^2. Unless specified $p-$value is less than 0.00001.

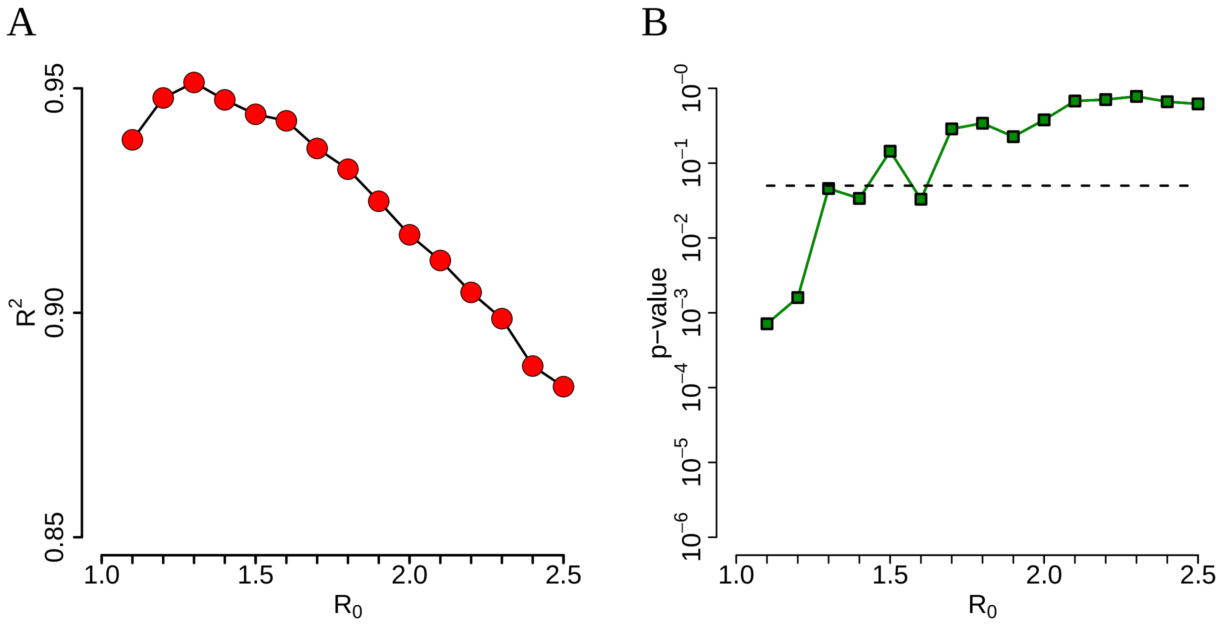

Figure S2: **A** Coefficient of determination R^2 of the linear model between outbreak probability and all parameters regulating the interventions, for different values of R_0. **B** Significance (p-value) in logarithmic scale of T_p variable in the linear model, for different values of R_0. Horizontal dashed line represent the significance threshold 0.05.

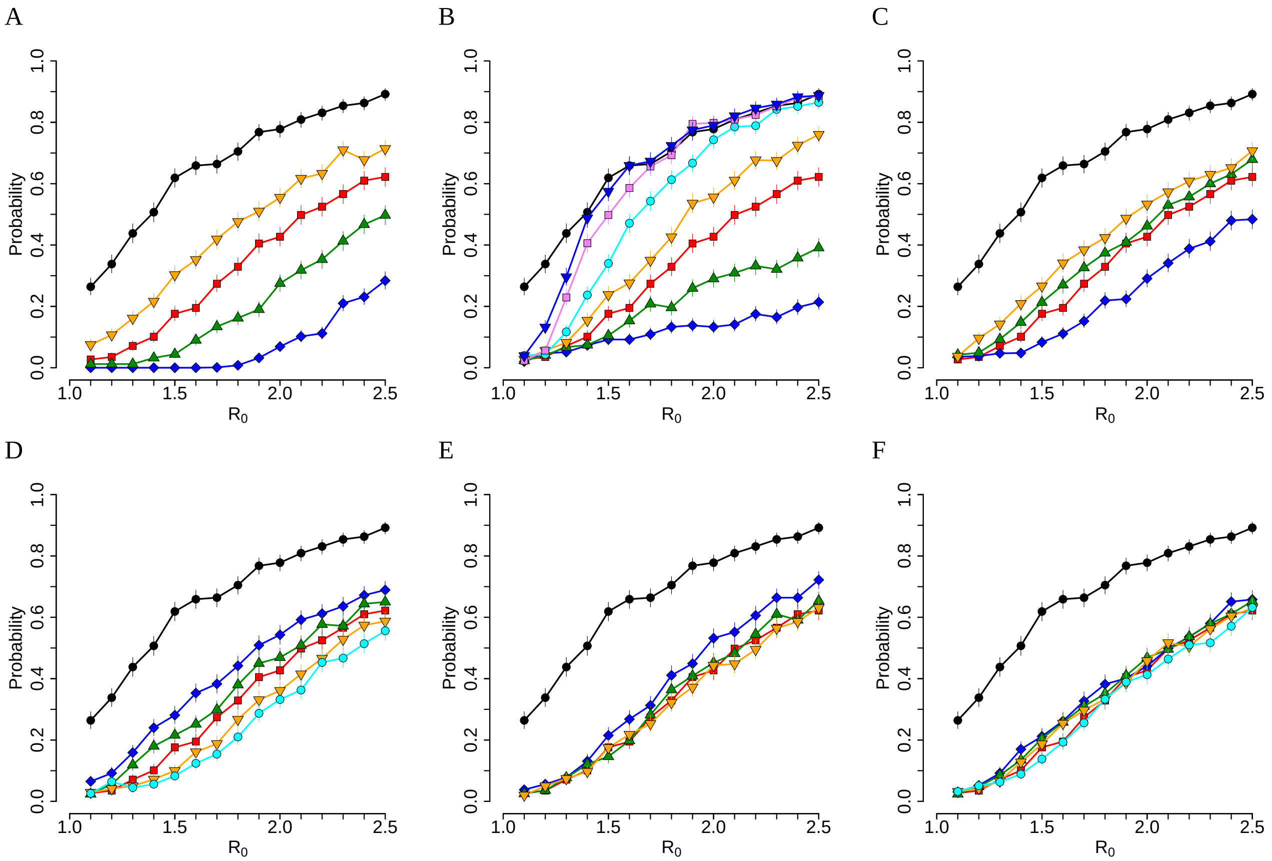

Figure S3: Probability of outbreak for different values of R_0 by assuming baseline scenario (uncontrolled epidemics, black), reference scenario (red) and by varying one-by-one the value of the following parameters. **A** Probability of detection of infected close contacts P_c: 1 (blue), 0.8 (green) and 0.4 (orange). **B** Delay from initial warning to intervention T_i: 0 (blue diamonds), 1 (green), 5 (orange), 10 (light blue), 20 (pink), 30 (blue triangles). **C** Delay from case detection to household quarantine T_t: 0 (blue), 2 (green) and 4 (orange). **D** Probability of detection of infected random contacts P_g: $0.9P_c$ (light blue), $0.7P_c$ (orange), $0.3P_c$ (green) and $0.1P_c$ (blue). **E** Different policy of intervention: contact tracing, laboratory closure, quarantine of laboratory workers households and closure of schools and workplaces attended by households members of laboratory workers (orange), contact tracing and laboratory closure (green) and only contact tracing (blue). **F** Self reporting probability P_r: $0.9P_g$ (light blue), $0.7P_g$ (orange), $0.6P_g$ (green) and $0.5P_g$ (blue).

of epidemics can spread without ascertainment of the virus escape (see also Figure 2 of the Main Text).

The other most relevant parameter is T_i. Figure S3B clearly shows that delay in the start of intervention drastically increases outbreak probability: in particular starting interventions with a delay of more than 20 days for epidemics having R_0 larger than 1.3 is almost equivalent to not intervening at all. On the other hand, starting interventions with a delay of 1 day only can drastically increase the containment probability even for large values of R_0.

Figure S3C shows that while a delay of 2 days in household quarantine after case detection does not make a large difference, a delay of 4 days can make containment much more difficult, even for low values of R_0; on the other hand, household quarantine immediately after case

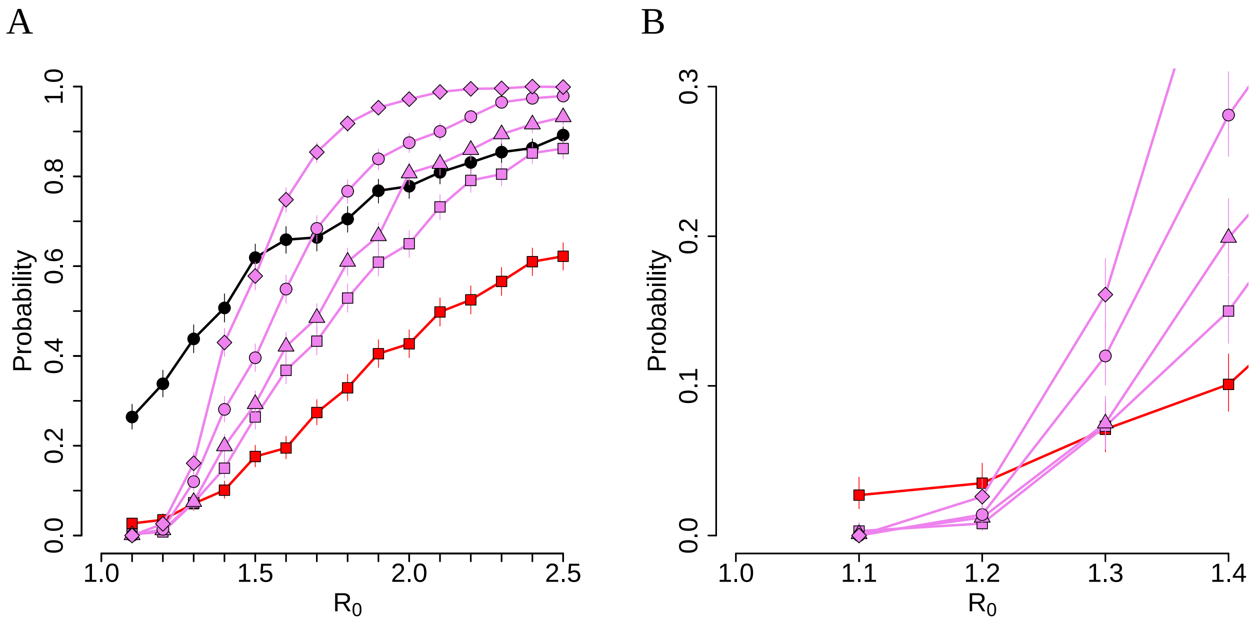

Figure S4: **A** Probability of outbreak for different values of R_0 by assuming baseline scenario (uncontrolled epidemics, 1 initial case, black line), and reference scenario under different number of index cases: 1 case (red line), 2 cases (pink squares), 3 cases (pink triangles), 5 cases (pink dots) and 10 cases (pink diamonds). **B** Zoom of the bottom left part of panel **A**.

detection, though very difficult to implement, would lead to systematically better results in terms of outbreak containment.

Figure S3D shows that the variability in P_g has a clear impact on outbreak probability.

Figure S3E shows the effect of different initial interventions on the laboratory's contact network. It shows that closure of the laboratory is the only crucial intervention to put in place soon after detection of the accidental escape, especially for large values of R_0. Additional measures such as quarantine of laboratory workers households and closure of schools and workplaces attended by households members of laboratory workers are of little impact.

Figure S3F shows the negligible impact of changes in P_r. This is due to the fact that changing P_r from $0.5P_g$ to $0.9P_g$ means changing P_r from 0.15 to 0.27.

2.3 Different number of index cases

By assuming a larger number of index cases among laboratory workers, containing the epidemic becomes more difficult (see Figure S4A). However, increasing the number of index cases also increases the probability of detecting the escape from the BSL facility; this effect is largely appreciable for $R_0 = 1.1$ and 1.2, in fact by assuming 2-5 index cases in the laboratory, the epidemic outbreak probability when the reference intervention is assumed is nearly 0 (see Figure S4B).

2.4 Different seeding locations

Results reported in the main text mainly refer to the accidental release from a BSL facility located in Rotterdam (NL). In order to understand whether our findings may be affected by population density and country-specific socio-demography, we consider alternative locations for

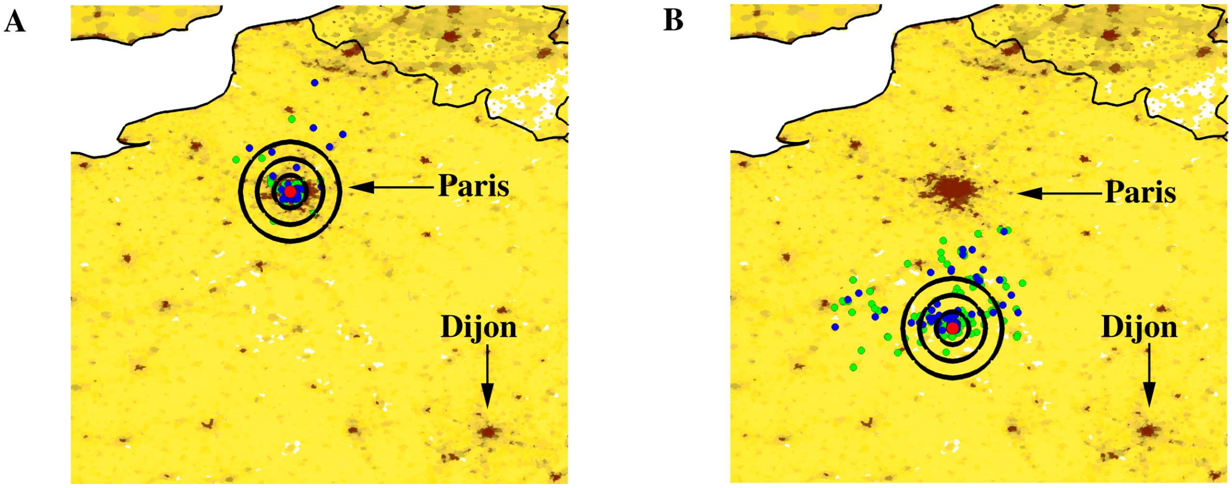

Figure S5: **A** The map shows population density of a given area of France (colors from yellow to dark brown indicate increasing densities, scale as in Figure 1 of the main text), the location of the BSL facility (in Paris, red point), the location of workers houses (blue points), the location of workplaces and schools attended by household members of laboratory's workers (green) in one randomly chosen simulation. Black concentric circles indicate distances of 10km, 20km, 30km from the laboratory. **B** As **A** but the BSL facility is located in a rural area south of Paris.

the BSL facility, located both in urban (London, UK, Stockholm, SE, Milan, IT, Madrid, ES, Paris, FR) and rural areas. Rural areas are low population density areas – small towns or villages – in Wales (UK, ≈ 80 km north of Cardiff), Uppland (SE, ≈ 100 km north of Uppsala), Sardinia island (IT, ≈ 50 km east of Sassari), Andalusia – Castile la Mancha (ES, ≈ 50 km north-east of Cordoba), Centre-Burgundy (FR, ≈ 80 km south-east of Orleans). We do not consider further locations for the BSL facility in the Netherlands, as population density is rather homogeneous in the whole country and it is difficult to identify wide low-density areas. In the main text we compare results by assuming reference interventions in all seeding areas (see Fig. 4 in the main text). Here we analyze results for France, by considering both urban and rural seeding (see Figure S5A and B), in greater detail.

First of all, we consider the case where the BSL facility is located in Paris. Without considering control measures, the probability of observing an epidemic outbreak after virus escape is quite similar to that in the Dutch scenario: slight differences can be observed for low values of R_0 (see Figure S6A). On the other hand, marked differences exist when the reference intervention is put in place: in particular for R_0 values lower than 1.7 outbreak probability in the Paris scenario is more than two times higher than in the Netherlands case (see Figure S6A). Such large differences can be due to dissimilarities in social and demographic characteristics of French and Dutch populations because, despite a general similarity, some marked country-specific features such as age structure and average household size exist (see the comparison presented in [1]). However, although quantitatively different, the general patterns obtained by varying P_c and T_i are the same observed in the Dutch case (compare Figure S6B and C with Figure S3A and B).

Second, we discuss the case where the BSL facility is located in a low-density area (≈ 80 km south-east of Orleans, 150 km south of Paris). As shown in Figure S5, the commuting distance traveled by individuals in the low-density area is larger than that observed for urban areas; this pattern is consistent with what has been reported in [5]. As we assume the same social and demographic characteristics of the population in the whole France, by comparing this

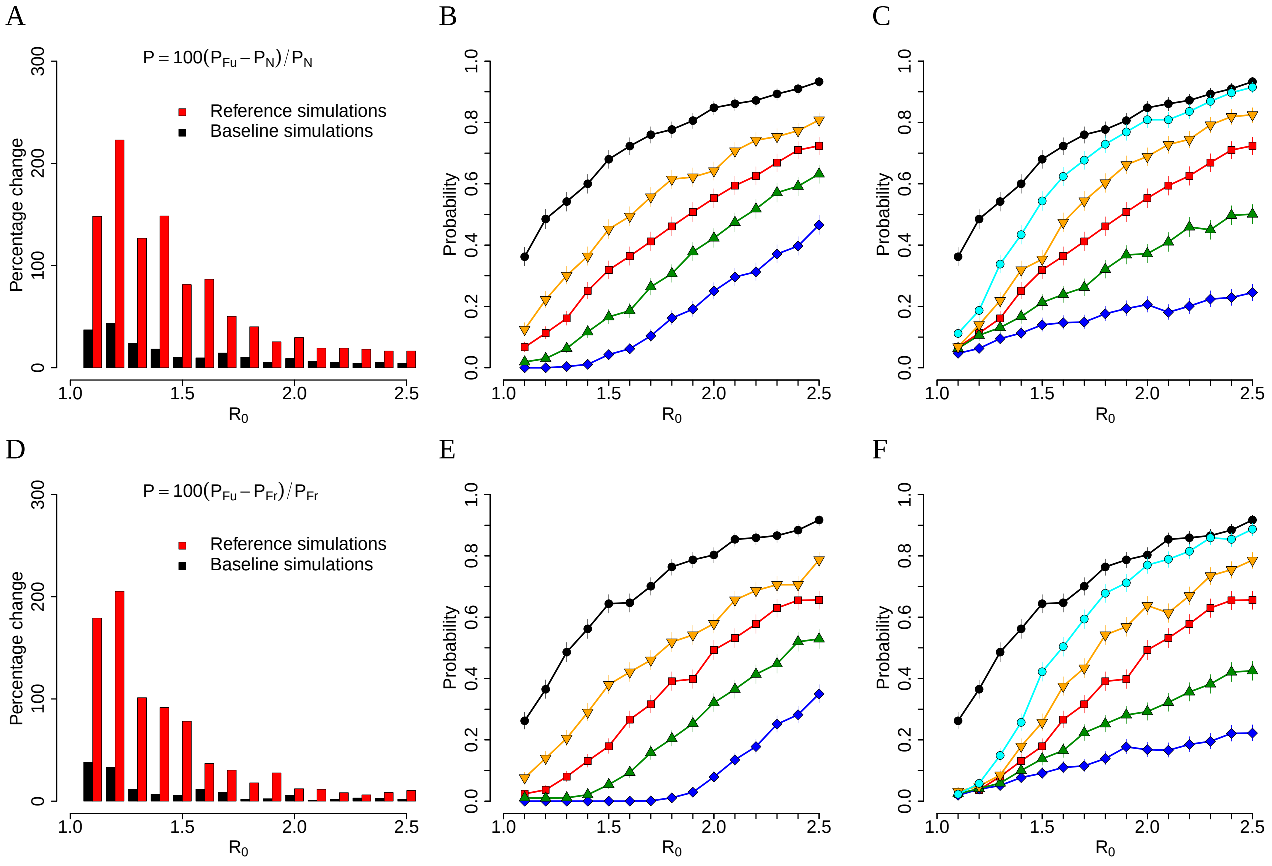

Figure S6: **A** Percentage variation of the outbreak probability of simulations where the BSL facility is located in Paris, France (subscript F_u in the formula) with respect to simulations having seeding in Rotterdam, The Netherlands (subscript N), for different values of R_0, for both baseline (black) and reference (red) scenarios. **B** Probability of outbreak for simulations seeded in Paris for different values of R_0 by assuming baseline scenario (uncontrolled epidemics, black), reference scenario (red) and for different values of P_c, namely: 1 (blue), 0.8 (green) and 0.4 (orange). **C** Probability of outbreak for simulations seeded in Paris for different values of R_0 by assuming baseline scenario (uncontrolled epidemics, black), reference scenario (red) and for different values of T_i, namely: 0 (blue), 1 (green), 5 (orange) and 10 (light blue). **D** Percentage variation of the outbreak probability of simulations where the BSL facility is located in a rural area south of Paris (subscript F_r) with respect to simulations having seeding in Paris, for different values of R_0, for both baseline (black) and reference (red) scenarios. **E** As **B**, but the simulations are seeded in a rural area south of Paris. **F** As **C**, but the simulations are seeded in a rural area south of Paris.

case with the Paris one, we can appreciate the net effect of different population densities. From the comparison shown in Figure S6D, outbreak probabilities for untreated epidemics are similar, while a largely different effect of containment measures can be observed: in particular, outbreaks starting in densely populated areas are more difficult to control. This is also clear by looking at the effectiveness of interventions as obtained by varying P_c and T_i (see Figure S6E and F).

References

[1] Fumanelli L, Ajelli M, Manfredi P, Vespignani A, Merler S (2012) Inferring the Structure of Social Contacts from Demographic Data in the Analysis of Infectious Diseases Spread. PLOS Comput Biol 8: e1002673.

[2] Merler S, Ajelli M (2010) The role of population heterogeneity and human mobility in the spread of pandemic influenza. Proc R Soc B 277: 557–565.

[3] Merler S, Ajelli M, Pugliese A, Ferguson NM (2011) Determinants of the Spatiotemporal Dynamics of the 2009 H1N1 Pandemic in Europe: Implications for Real-Time Modelling. PLOS Comput Biol 7: e1002205.

[4] Gonzalez MC, Hidalgo CA, Barabasi AL (2008) Understanding individual human mobility patterns. Nature 453: 779–782.

[5] Ferguson NM, Cummings DA, Cauchemez S, Fraser C, Riley S, et al. (2005) Strategies for containing an emerging influenza pandemic in Southeast Asia. Nature 437: 209-214.

[6] Ferguson NM, Cummings DAT, Fraser C, Cajka JC, Cooley PC, et al. (2006) Strategies for mitigating an influenza pandemic. Nature 442: 448–452.

[7] Ciofi degli Atti ML, Merler S, Rizzo C, Ajelli M, Massari M, et al. (2008) Mitigation measures for pandemic influenza in Italy: an individual based model considering different scenarios. PLOS ONE 3: e1790.

[8] Cauchemez S, Bhattarai A, Marchbanks TL, Fagan RP, Ostroff S, et al. (2011) Role of social networks in shaping disease transmission during a community outbreak of 2009 H1N1 pandemic influenza. Proc Natl Acad Sci USA 108: 2825–2830.

[9] Cauchemez S, Carrat F, Viboud C, Valleron AJ, Boëlle PY (2004) A Bayesian MCMC approach to study transmission of influenza: application to household longitudinal data. Stat Med 23: 3469–3487.

[10] Cauchemez S, Valleron AJ, Boëlle PY, Flahault A, Ferguson NM (2008) Estimating the impact of school closure on influenza transmission from Sentinel data. Nature 452: 750–754.

[11] Kwok KO, Leung GM, Riley S (2011) Modelling the Proportion of Influenza Infections within Households during Pandemic and Non-Pandemic Years. PLoS ONE 6: e22089.

[12] Anderson RM, May RM (1992) Infectious diseases of humans: dynamics and control. Oxford, UK: Oxford University Pres.

[13] Wallinga J, Lipsitch M (2007) How generation intervals shape the relationship between growth rates and reproductive numbers. Proc R Soc B 274: 599–604.

[14] Longini IM, Nizam A, Xu S, Ungchusak K, Hanshaoworakul W, et al. (2005) Containing pandemic influenza at the source. Science 309: 1083-1087.

[15] Carrat F, Vergu E, Ferguson NM, Lemaitre M, Cauchemez S, et al. (2008) Time lines of infection and disease in human influenza: a review of volunteer challenge studies. Am J Epidemiol 167: 775–785.

[16] Balcan D, Colizza V, Gonçalves B, Hu H, Ramasco JJ, et al. (2009) Multiscale mobility networks and the spatial spreading of infectious diseases. Proc Natl Acad Sci USA 106: 21484–21489.
